# A scoping review of the social dimensions in food insecurity and poverty assessments

**DOI:** 10.3389/fpubh.2022.994368

**Published:** 2022-12-22

**Authors:** Tina Bartelmeß, Sarah Jasiok, Elias Kühnel, Juliane Yildiz

**Affiliations:** ^1^Faculty of Life Sciences: Food, Nutrition, and Health, University of Bayreuth, Kulmbach, Germany; ^2^Faculty of Law and Economics, University of Bayreuth, Bayreuth, Germany; ^3^Faculty of Agricultural, Nutritional and Environmental Sciences, Justus-Liebig-University Giessen, Giessen, Germany

**Keywords:** food poverty, food security, food insecurity, social dimensions, indices, indicators

## Abstract

Food poverty is a phenomenon that is currently receiving increasing social attention in both the Global South and the Global North. It is often equated with food insecurity, for which numerous assessment tools and reports exist. However, only limited specific data can be found on food poverty. Starting from a theoretical concept of food poverty, this article examines in a scoping review which dimensions of food poverty are captured by indices and indicators of food insecurity and general poverty assessments. The review focuses particularly on the social dimension of food poverty and points out that it is under-reported and that no adequate assessment tools exist so far. Existing indices and indicators of food insecurity and general poverty assessments are critically reviewed and suggestions for the assessment of social food poverty for policy and practice derived.

## 1. Introduction

Food insecurity and poverty are currently once again at the center of socio-political attention both in countries of the Global South and the Global North. Weather extremes, recent conflicts such as the Russo-Ukrainian war and associated economic shocks are tipping the global food system and endangering food supplies and individual livelihoods (1). In 2020 with the outbreak of COVID-19 numerous global institutions, such as the FAO or the United Nations, warned of a global food crisis. By 2022 at the latest, this global food crisis manifested and intensified further with an unprecedented global hunger crisis and 345 million people globally affected by acute food insecurity (2). Acute or transitory food insecurity refers to “a sudden (and often precipitous) drop in the ability to purchase or grow enough food to meet physiological requirements for good health and activity” [(3), p. 1]. It differs from chronic food insecurity, which currently affects 828 million people worldwide (2), in that it is not long-term and persistent, but rather short-term and temporary. However, chronic, and transitory episodes of food insecurity can overlap, so that people can slip from acute food insecurity into chronic insecurity in the long term (4, 5). It is therefore essential to detect transitory food insecurity at an early stage to take measures to prevent people from deteriorating into chronic food insecurity.

The number of chronically as well as transitory food insecure households is significantly higher in the Global South, currently especially in high-concern hotspots like African and Arab countries, than in the Global North (6). Causes for the uneven geographical distribution of food insecurity are attributed, among other things, to structural inequalities in terms of political stability, economic security, environmental events, and access to health services (7, 8). But food insecurity is also a phenomenon currently on the rise in the Global North and especially in Europe. Data based on the FAO Food Insecurity Experience Scale (FIES) indicate prevalence rates ranging from as low as 3.1% in some countries to more than 20% in other European countries (9, 10). Even though prevalence rates seem to be low in some European countries, increasing numbers of people using food banks since the outbreak of the COVID-19 pandemic indicate a growing acute food insecurity problem (11). Across Europe, the consumer price index and the cost of living and energy are rising, all of which affect the affordability of food for households (12, 13). However, food insecurity does not persist to the same extent in all European countries, as shown by the wide range of FIES prevalence rates and the varying prominence of food banks in different countries (10). It appears that food insecurity in Europe does not manifest itself in the same facets as in some countries of the Global South, where high rates of chronic or acute food insecurity have been detectable for decades and can be captured by existing measurement approaches. At present, it is not possible to say much about the precise number of households in Germany who are dependent on food from the food banks due to a lack of official statistics (14). However, it is already known that the use of food banks is often accompanied by shame and a feeling of discrimination and that the actual use of food banks is often only a late manifestation of food insecurity in Germany (15, 16).

Since food insecurity has so far mainly been treated as a phenomenon of the Global South, there is hardly any differentiated data on the status of food insecurity at the level of European countries. When searching for current data on food insecurity in Germany, for example, only the latest FAO data from 2020 on severe food insecurity can be found. With a prevalence rate of 1.1% and an increase of 0.4 percentage points since 2019 (10), these data indicate that severe food insecurity is also present in Germany, with a tendency to rise. However, the data tell us nothing about the different and probably varying degrees and manifestations of food insecurity as distinct from countries in the Global South. At this level, only data collected through the FIES indicator questionnaire is used to determine whether an adult in the household reported having been exposed at times during the year to several of the severe experiences described in the FIES indicator questions, such as being forced to reduce the amount of food, skip meals, go hungry, or not eat for an entire day due to lack of money or other resources (10). While the FIES assessment is already experienced-based, it relates specifically to the dimension of access to food and related challenges in the Global South. In this respect, the coverage is heavily biased toward international comparisons of levels of severe food insecurity, which seem to be largely mitigated in the Global North by institutions such as food banks. Severe levels of food insecurity are likely to result in lower food intake and can therefore lead to more severe forms of malnutrition, including hunger (17), which have not yet occurred on such a large scale in the Global North. Moderate levels of food insecurity, on the other hand, are usually associated with the inability to eat a healthy and balanced diet on a regular basis (18). Therefore, a high prevalence of moderate-level food insecurity can be considered a predictor of various forms of diet-related health impairments in the population that are associated with micronutrient deficiencies and unbalanced diets (18). In the case of Germany, evidence for precisely these manifestations of food insecurity is regularly provided by representative population-based nutrition and health surveys (19–21). In these population surveys, socioeconomic status (SES) is usually considered an independent variable to explain dietary and health behavior in relation to social inequalities. Results have repeatedly shown that people with low SES tend to eat unhealthier and are more likely to be affected by lifestyle-related diseases (21–23). But even health and nutrition reporting in Europe and Germany in particular reveals little about how food insecurity manifests itself in the Global North beyond nutrition and health behavior. It is already known that when household incomes are reduced, people tend to reach for cheaper, usually high-sugar and high-fat, foods and tend to “stretch” meals (22, 24). Apart from the physiological consequences of food insecurity and the way socio-economically disadvantaged households deal with food, we know that also in the Global North, food and eating take on important socio-cultural functions. In sociological terms, food is one of the most important means of establishing identity, demarcation, and community (25–27). Food behavior research has shown that when household incomes are reduced, the first sacrifice is participation in social occasions in that food often plays a major role (27, 28). As Dowler states: “Food is an expression of who a person is and what they are worth, and of their ability to provide their family's basic needs; it is also a focus for social exchange (…) But it is not just health that is compromised in food-poor households: social behavior is also at risk” [(29), p. 58]. In Europe, poverty reporting is partially dedicated to these socio-cultural deprivations, also in relation to food. For example, the European Union Statistics on Income and Living Conditions (EU-SILC) assesses poverty risks in different areas of life and asks whether households can afford an occasional meal in a restaurant when assessing the degree of poverty risk. In poverty reporting, however, food and nutrition are only one area of life, and it cannot be assumed that it is covered in a more differentiated way. Poverty research and reporting in Germany currently reports alarming levels. In 2021, the at-risk-of-poverty rate in Germany was 16.6% (30). Accordingly, almost one-fifth of Germany's population is affected by income poverty. Comparing food insecurity and poverty coverage, it is hard to imagine only a small proportion of people affected by poverty are also affected by food insecurity.

Given the theoretical considerations that socio-cultural deprivation in food and nutrition can be seen as early indicators of impending food insecurity (27, 31) and that greater prevalence of severe food insecurity in the Global North cannot be demonstrated despite apparent societal observation, the question arises whether and to what extent aspects of socio-cultural deprivation in food and nutrition have so far been assessed representatively. This article provides an overview of the indices and indicators used to assess food insecurity and poverty and explores the extent to which sociocultural aspects of food insecurity or poverty are captured. We draw on a sociological model of food poverty that helps identify and systematize the indices and indicators along different social dimensions (32). The questions the review seeks to answer are, first, which dimensions of food deprivation are captured by the indices and indicators of food insecurity and poverty, and second, how the sociocultural dimensions of food deprivation are reflected in the indices and indicators identified. The article is structured as follows. In section two, we will present the sociological model of food poverty, which provides us with evidence on sociocultural dimensions of food deprivation and serves as a starting point for systematizing the indices and indicators. In section three, we will explain our approach to identifying the indices and indicators. In section four we will present the results and section five follows the discussion. We conclude with limitations of the review, and recommendations for further research.

## 2. A theoretical model of (social) food poverty

While food (in)security is a concept that has been defined numerous times by scholars and supranational institutions, and where the definition of the FAO has meanwhile gained acceptance (17, 33), food poverty is a concept that has so far been insufficiently defined (34). The term food poverty is often used interchangeably with food insecurity. Food insecurity historically addresses the more complex interrelationships between, among other things, physical and hygienic availability, and economic access to food at the national level (18). In the meantime, however, the term household food insecurity has emerged to refer to the fact that access, availability, and utilization of food are to a considerable extent condemned by financial constraints at the household level. A whole range of factors have been identified, such as education, food aid, income, gender, etc., and associations with the three dimensions of availability, access and utilization at the household level have been demonstrated (35). The measurement of household food insecurity focuses on households' self-reports of insecure, inadequate, or inappropriate access to food, its availability and utilization resulting from limited financial resources, and the subsequently restricted food consumption and eating habits (36). The measurement is thus based on an understanding of changes in food as a marker of material household deprivation. Household food insecurity reflects the inability of households to afford food as a basic need due to a lack of financial resources and serves as an important indicator of poverty. Hence, what is captured under household food insecurity can be described as material food poverty. Following a food poverty model developed by a German research group in the 1990s (28, 37, 38), material food poverty is segmented into four sub-dimensions. First, the economic dimension refers to the lack of financial resources to access food. Second, the physical dimension refers to physical access to food or utilities. Third, is the physiological dimension, which refers to inadequate nutrient and energy supply and low nutritional quality of food. Fourth, is the hygienic dimension, which refers to the availability of hygienically safe food. Based on Townsend et al. (39) fundamental distinction between material and social deprivation, the material dimensions of the food poverty concept are indeed still relatively equivalent to those of food insecurity, however, for Townsend deprivation is also manifested in social dimensions: “Non-participation in the roles, relationships, customs, functions, rights and responsibilities implied by membership of a society and its sub-groups” [(39), p. 36] is closely linked to material food deprivation and usually occurs simultaneously. Basic needs, such as nutrition, are tied to social functions. For example, the act of visiting friends where one is served a drink is linked to not only nutritional but also social needs (40–42). The foods people turn to are strongly linked to their identities, and involuntary abstaining from them can lead to social exclusion (41). Based on the Townsend classification, Feichtinger (37) and Köhler et al. (28) developed a comprehensive concept of food poverty that includes social dimensions as well as material ones and includes a temporal component (see Table 1).

**Table 1:** Dimensions of food poverty [own adaption from (37), p. 9].

**Dimensions and sub-dimensions**	**Description**	**Examples of deprivation**
**Material dimensions**		
Economic	Financial means to purchase food	Impairment of participation in food consumption
Physical	Institutional and infrastructural food supply, self-sufficiency options	Lack of spatial access to food and supply services
Physiological	Supply with energy and nutrients	Impairment of mental and physical performance and health due to nutritional status
Hygienic	Supply and availability of safe food	Health impairment due to contaminated foods
**Social dimensions**		
Social	Social organization, integration and demarcation, social security, communication	Impairment of social relationships (e.g., when invitations can no longer be returned), social exclusion
Cultural	Normative value systems, food customs and traditions, edibility, taste	Deviation from socio-culturally accepted diets and eating habits
Mental	Pleasure, emotional security, compensation, self-esteem	Loss of self-affirmation, overcompensating to bizarre coping strategies
Temporal	Effects of frequency and duration of certain nutritional situations over time	Subsequent impacts in various subdimensions

Social food poverty denotes a diet “that does not enable people to form social relationships, assume roles and functions, exercise rights and responsibilities, or fulfill customs and traditions expressed in the social and cultural utilization of food in a society in a socially acceptable manner” [own translation, (37), p. 8]. The dimension of social food poverty, therefore, emphasizes the social, cultural, and mental (psychological) functions of food and nutrition as indicators of experienced deprivation or as causes of food poverty.

The social dimensions of food poverty recognize that food practices always include aspects of social participation, whether through celebrations like birthday parties, business meetings or encounters with friends. Food has a strong symbolic value that goes beyond the satisfaction of biological needs and is closely linked to identity, social relations, group affiliation and differentiation (25, 26, 43). Sociological conceptualizations of poverty also consider experiences of impoverishment in relation to lifestyles that might not be sustained due to various forms of deprivation (27). As such the socio-cultural sub-dimensions of food poverty are not just a subjective experience but also a social classification (27, 44) undertaken by oneself and others in relation to what food practices of a certain lifestyle are perceived as socio-culturally appropriate and no longer can be accomplished in deprived situations. Thus, the social sub-dimensions particularly refer to the notion that food and nutrition are essential mechanisms of socio-cultural participation and mental wellbeing (45). Suffering from food poverty can therefore go hand in hand with feeling socially excluded, not being able to follow cultural traditions and socio-cultural habits and the subsequent loss of mental health (24). The social dimensions of food poverty can be summarized as both conditional factors and consequential dimensions in which deprivation is expressed and experienced. The extent to which social dimensions of food poverty are captured in the practice of food (in)security and poverty assessment and what statements can be derived from the indicators about the manifestation of social food deprivation is the subject of the following considerations.

## 3. Materials and methods

Food insecurity and poverty reporting are usually based on representative population surveys that use indices or indicators to capture the status quo in a comparable and consistent way (46). Over the decades, indicators have been proposed to measure food insecurity and overall poverty levels, ranging from confined measures of specific variables (e.g., household income, percentage of undernourished children etc.) to complex indices that aim to summarize the multiple dimensions that characterize food insecurity or poverty (e.g., Global Food Security Index, World Hunger Index, Multidimensional Poverty Index, etc.) (47, 48). Based on the theoretical considerations in section two, indices and indicators used in reporting on food insecurity and general poverty are collected in a scoping review (49) and clustered according to the addressed dimensions of the concept of food poverty by Feichtinger (37).

### 3.1. Research questions

The scoping research questions were:

1. What dimensions of food poverty are covered by the identified indices and indicators?
2. How is social food poverty addressed in the identified indices and indicators?

### 3.2. Search strategy

In spring 2022 (March/April), we conducted two systematic web searches. The first search focused on indices and indicators used in food insecurity reporting. The second search focused on general poverty reports with the aim of extracting nutrition-related indicators in these reports. We used Google search engine to search for food insecurity indices and indicators, using keywords and various combinations. The search terms used can be classified in three dimensions: (1) nutrition-related terms (“food” or “nutrition”), (2) deprivation-related terms (“security”, “insecurity”, “poverty”, “deprivation”, and “wellbeing”) and (3) “index”, “indices” or “indicator”. We performed a total of 24 searches, each with different combinations of terms, and screened the first 100 hits and listed relevant indices and indicators until theoretical saturation was reached and no further relevant results could be identified. For searching food-related indicators in poverty reports, we manually searched the websites and reports of various international and national institutions and ministries [such as FAO, World Health Organization (WHO) or Organization for Economic Co-operation and Development (OECD) as well as Federal Ministries of Labor and Social Affairs in Germany and equivalents in other countries]. We started by searching for national poverty reports and poverty-related official documents in European Union (EU) countries, the United Kingdom (UK) and the United States (US) *via* a google search and the respective websites of the federal ministries. The reports and documents were identified by screening the websites of the ministries and institutions using the following terms: “poverty”, “deprivation”, “inequality”, “living conditions”, “food security”, “food insecurity”, “food”, and “nutrition”. We subsequently downloaded all available reports and manually searched for nutrition-related indicators by using nutrition-related keywords (e.g., food, diet, nutrition, and eat^*^). All hits that were considered relevant in the first and second search were entered into an Excel spreadsheet, separately for food insecurity and general poverty report index or indicator, supplemented with additional information [e.g., country or level of application, aggregation level (market, individual, household), sources], and in a second step screened in detail for the inclusion and exclusion criteria.

### 3.3. Inclusion and exclusion criteria

To be included in the scoping review, the indices and indicators had to describe at least one aspect that can be assigned to a dimension of food poverty according to Feichtinger's (37) concept. In addition, poverty reports in which indices and indicators were identified had to have a publication date between 2017 and 2022 and be in English or German. Indices or indicators were included if they had already been used to collect representative data for one of the European, UK, or US countries. Indices or indicators that were not in use and had not yet been considered or used on a trial basis were excluded.

### 3.4. Data extraction

Based on the excel spreadsheets with the final hits on food insecurity and poverty indices and indicators, the indices and indicators were examined in more detail, and it was determined which dimensions of Feichtinger (37) food poverty concept they are related to. For the indices whose indicators allowed statements about the social dimension or its sub-dimensions, the corresponding indices and topic aspects were recorded. Out of 142 food (in)security indices or indicators found, a total of 20 could be classified as relevant. From the Food Poverty Reports, the first step was to take stock of all the indices mentioned in the reports and to examine the extent to which food and nutrition were addressed. Food and nutrition-related indices and survey questions from the reports were then systematically compiled in an Excel spreadsheet according to the dimensions of Feichtinger (37). In total, 14 reports could be identified as relevant, two of which related to Germany, four to the UK, four to the US, three to the EU and one to the global level. In the final step, the indices, and indicators of the searches on food (in)security and food-related poverty were assigned to the food poverty dimensions in a synthesizing table, and the relevant indices and indicators identified were designated and outlined with regard to the food-related knowledge objects in the individual sub-dimensions (see Table 2).

**Table 2:** Food (in)security and poverty reports indices and indicators referring to food poverty dimensions.

**Dimension**	**Indicators**	**Description**	Food (in)security indices^*^	Poverty reports indices and indicators^**^
**Material food poverty**				
Economic	Food affordability	Food price levels, stability, and shocks, local cost of food required to meet a common energy intake	FAI; GFI; GFSI; UK-FSR	CPI
	Household-income	Lack of monetary resources to purchase food; share of income spent on food	CARI; FEI; FIES	MD; MSD
	Unconventional food income source strategies	Coping strategies to get money for food	CSI; FSS; LCS-FS	
Physical	National availability of food	Food supply, supply disruption, domestic production (export), food import dependence, food loss/waste; available food diversity/quality	Food-EPI; FSI; GFSI; HEI; MLDS	
	Food environment	Proximity to grocery stores; access to a reliable food/water source	FEI; GFSI; UK-FSR	MDI; MPI
	Food distribution	Quality of the road infrastructure	FSI	
Physiological	Malnourishment	Prevalence of undernourishment (stunting, wasting), overnutrition	FSI; GHI; GFI	BMI; MPI
	Diet quality	Healthy and nutritious food, dietary diversity, adequacy of micronutrient intake, consumption levels	DQI-I; FIES; Food-EPI; FSI; GFI; GFSI; HDDS; HEI; HFIAS	
	Food quantity	Consumption of different food groups, situations of hunger	CARI; FCS; HHS	
	Diet-related health outcomes	Diabetes, obesity, disability-adjusted-life-years, mortality rates	FSI; GFI; GHI	MPI; SPI
Hygienic	Food safety	Access to safe food and drinking water	GFI; GFSI	MPI; MPM; SPI
**Social food poverty**				
Social	Social integration	Getting together, eating out		MSD
	Communal networks	Coping behaviors (e.g., sending children to eat with neighbors); unconventional food sources (e.g., borrowing food from neighbors)	CARI; CSI; LCS-FS	
	Social food access barriers	Gender inequality in household food access, free institutional meals	CSI; FSS; GFSI; UK-FSR	
Cultural	Food customs and practices	Deviant food patterns, dietary change	CARI; CSI; FSS; HFIAS; HHS	FRS; MD; MSD
Mental	Worries about food	Uncertainty or concerns about insufficient food procurement	FIES, HFIAS; LCS-FS	FRS
	Bizarre coping strategies	Illegal income activities (theft, prostitution) due to lack of food, begging or scavenging for food	LCS-FS	

* CARI, consolidated approach for reporting indicators of food security; CSI, coping strategy index; DQI-I, diet quality index-international; FAI, food affordability index; FCS, food consumption score; FEI, food environment index; FIES, food insecurity experience scale; Food-EPI, food environment policy index; FSI, food sustainability index; FSS, food security supplement; GFI, global food index; GFSI, global food security index; GHI, global hunger index; HDDS, household dietary diversity scale; HEI, healthy eating index; HFIAS, household food insecurity access scale; HHS, household hunger scale; LCS-FS, livelihood coping strategies-food security; MLDS, market-level food diversity score; UK-FSR, UK-food security report indicators.

** BMI, body mass index; CPI, consumer price index; FRS, UK family resource survey indicators; MD, material deprivation indicators; MDI, multidimensional deprivation index; MPI, multidimensional poverty index; MPM, multidimensional poverty measure indicators; MSD, material and social deprivation indicators; SPI, social progress index.

## 4. Results

### 4.1. Dimensions of food poverty covered by identified indices and indicators

The review aimed to identify indices and indicators that are used in food insecurity and poverty assessments and to shed light on the dimensions of food poverty that are covered by these indices and indicators.

In recent decades, many food (in)security indices and related indicators have been developed and applied {e.g., Global Food Security Index (GFSI), Good-Enough-To-Eat-Index [also named Global Food Index (GFI)] or Global Hunger Index (GHI)}. These indices focus on different dimensions of the food (in)security concept (e.g., availability, access, and utilization) and aim to monitor the status quo and progress in food (in)security in an internationally comparable manner. As can be seen in Table 3, when transferring the indices used in food (in)security measurements to the dimensions of food poverty, most of these indices focus on material food poverty. An important factor influencing their applicability and informative value in relation to the food poverty concept is that the respective indicators largely refer to the level of entire nations and rely on data available at this level. Of the 20 indices identified in official reports on food (in)security, ten include indicators that can be assigned to the economic dimension, seven to the physical dimension, 15 to the physiological dimension, and two include indicators that can be assigned to the hygienic dimension of food poverty. The economic and physiological dimension of food poverty are thus the dimensions that are most frequently assessed. In comparison to social food poverty, which is considered by a total of nine indices, it becomes clear that the focus of the measurement of food (in)security is on the indicators that can predominantly be used to describe the status of material food poverty. Of the nine indices that also have indicators for social food poverty dimensions, six include indicators for the social dimension, five for the cultural dimension, and three for the mental dimension of social food poverty.

**Table 3:** Food (in)security indices with indicators on food poverty dimensions.

	**Food (in)security indices**		**Material food poverty**				**Social food poverty**		
**No**	**Index/indicator**	**Name**	**Economic**	**Physical**	**Physiological**	**Hygienic**	**Social**	**Cultural**	**Mental**
1	CARI	Consolidated approach for reporting indicators of food security	x		x		x	x	
2	CSI	Coping strategy index	x		x		x	x	
3	DQI-I	Diet quality index-international			x				
4	FAI	Food affordability index	x						
5	FCS	Food consumption score			x				
6	FEI	Food environment index	x	x					
7	FIES	Food insecurity experience scale	x		x				x
8	Food-EPI	Food environment policy index		x	x				
9	FSI	Food sustainability index		x	x				
10	FSS	Food security supplement	x				x	x	
11	GFI	Global food index	x		x	x			
12	GFSI	Global food security index	x	x	x	x	x		
13	GHI	Global hunger index			x				
14	HDDS	Household dietary diversity scale			x				
15	HEI	Healthy eating index		x	x				
16	HFIAS	Household food insecurity access scale			x			x	x
17	HHS	Household hunger scale			x			x	
18	LCS-FS	Livelihood coping strategies-food security	x		x		x		x
19	MLDS	Market-level food diversity score		x					
20	UK-FSR	United Kingdom food security report 2021: theme 4: food security at household level	x	x			x		
***Σ***			10	7	15	2	6	5	3

Comparatively, in the poverty reports of European countries, the UK and the US, food-related indices and indicators are not covered more comprehensively in terms of both material and social food poverty. However, it should be noted that none of the identified reports commits to a definition of food poverty. Specific food poverty indices are also not mentioned and applied, as there exists no official food poverty indices. Rather, some reports refer to Townsend's distinction between material and social deprivation and see food as an area of life where social deprivation can be caused by material deprivation. Accordingly, all 14 identified poverty reports capture the economic dimension of material food poverty (see Table 4). Physiological dimensions are referred to in five poverty reports, but often in the form of self-reported time spent hungry due to a lack of financial means to obtain food (e.g., in the UK-Family Resource Survey). Characteristics of the physical and hygienic dimensions of material food poverty are recorded in a total of four reports in different combinations. In social food poverty, the scope of coverage is also low in the poverty reports. Only five of the 14 reports address the social sub-dimension, seven reports address the cultural sub-dimension and two reports the mental sub-dimension of social food poverty.

**Table 4:** Poverty reports with food-related indices/indicators.

	**Poverty reports**		**Material food poverty**				**Social food poverty**		
**No**	**Country**	**Name**	**Economic**	**Physical**	**Physiological**	**Hygienic**	**Social**	**Cultural**	**Mental**
1	Germany	Life Situations in Germany. The German Federal Government's Sixth Report on Poverty and Wealth 2021	CPI		BMI, MD			MD	
2	Germany	LEBEN IN EUROPA. Einkommen und Lebensbedingungen in Deutschland und der EU 2019	MSD		MSD		MSD	MSD	
3	UK	Living standards, poverty and inequality in the UK: 2021	CPI						
4	UK	UK Poverty 2022	CPI; FRS		FRS			FRS	FRS
5	UK	Poverty in the UK: statistics 2021	CPI						
6	UK	Family Resources Survey 2022	FRS		FRS			FRS	FRS
7	US	Income and Poverty in the United States: 2020	CPI						
8	US	Poverty in the United States in 2019	CPI						
9	US	Multidimensional Deprivation in the United States: 2017	CPI	MDI					
10	US	US the supplemental poverty measure 2020	CPI						
11	EU	Improving the understanding of poverty and social exclusion in Europe 2021	MSD	MPI	MPI; MSD; SPI	MPI; SPI	MSD	MSD	
12	EU	Living Conditions in Europe 2018	HICP; MSD		MSD		MSD	MSD	
13	EU	Monitoring social inclusion in Europe 2017	HICP; MSD	MPI	MPI; MSD	MPI	MSD	MSD	
14	Global	Poverty and Shared Prosperity 2020	CPI			MPM			
***Σ***			14	3	5	3	5	7	2

This quantitative overview reveals that the material deprivation is already recorded multi-dimensionally in the food (in)security indices and indicators, considering various conditional factors, which can be economic, physical, physiological, or hygienic. The poverty reports mainly consider the economic sub-dimension of material food poverty. Physical, physiological, and hygienic sub-dimensions play a subordinate role and the reports usually do not contain representative statistical data in this regard. However, the purely quantitative comparison of the coverage of dimensions of the food poverty concept by food (in)security indices and indicators and those used in general poverty reports already indicates that social deprivation in food and nutrition has not yet been comprehensively addressed. While the indices on food (in)security, in purely quantitative terms, mainly cover the social sub-dimension, the indices and indicators used in the poverty reports more often refer to the cultural sub-dimension. The next section describes the characteristics of social food poverty as outlined by the respective indices and indicators in the field of food (in)security and poverty assessment.

### 4.2. Social dimensions of food poverty in food (in)security and poverty assessments

*Food (in)security indices and indicators* that refer to dimensions of social food poverty leave the impression that the *social sub-dimension* of food poverty is determined by social access to food. They do not include aspects like, for instance, areas in social life where food deprivation might have social implications. In total, six indices could be identified that show references to the social dimension of food poverty. The Coping Strategy Index (CSI) and Consolidating Approach for Reporting Indicators of Food Security (CARI) assess, for example, whether households access food through community networks, such as borrowing food or seeking help from friends or relatives, or whether they send household members to eat elsewhere, such as with neighbors. Additionally, the Food Security Supplement (FSS) considers social access barriers and reflects whether households have access to price reduced or free school meals. Indices, such as the Global Food Security Index (GFSI) and CSI, also focus on social norms and the associated unequal distribution of social access to limited food resources in households, such as gender imbalances. The analysis of the indices revealed that five indices have references to the *cultural sub-dimension* of social food poverty. For example, CARI considers whether, in times of material food deprivation, people deviate from cultural dietary patterns, consume fewer meals than usual, or must adjust portion sizes. The FSS also takes into account whether preferred foods can still be consumed. However, the deviation from culturally accepted dietary patterns is only considered in terms of quantity and self-assessed preferences. There is no specification of prevailing cultural dietary practices and lifestyles, which implies that it is also not possible to record the extent to which culturally customary dietary practices must be refrained from in the everyday lives of vulnerable individuals and households. Consequently, it is not clear how non-participation in cultural food practices affects social exclusion and mental health, and how the different dimensions interact with each other. Furthermore, the *mental sub-dimension* of social food poverty is only addressed by three indices. The Livelihood Coping Strategies—Food Security Index (LCS-FS) records bizarre coping strategies to obtain food, such as begging for food or prostitution. The Household Food Insecurity Access Scale (HFIAS) and FIES include the item of worrying about having enough food.

It is evident that it is mainly manifestations of the individual sub-dimensions of social food poverty that are measured, which are easy to quantify and can be recorded cross-culturally. Specific social inequalities in social access to food are usually not addressed in food (in)security indices and indicators, nor are specific population groups and their socio-cultural characteristics explicitly addressed in previous indices of food (in)security. The Food Environment Policy-Index (Food-EPI) is an exception, as it addresses aspects of social inequality in relation to food and nutrition. However, in the Food-EPI social inequality is only analyzed in terms of material access to food, to healthy nutrition options, and corresponding health outcomes (50). Social dimensions of food poverty are not covered by the Food-EPI index, which was developed in Germany and is not yet widely applied. However, it is unique in capturing inequality between social groups, as well as relevant social-environmental and economic determinants of nutrition and health.

The seven *poverty reports* that refer to dimensions of social food poverty draw a similar picture of the phenomenon, as they use identical or similar indices or indicators to capture the individual sub-dimensions. In total, there are three indices or sets of indicators used to capture the characteristics of social food poverty. The Material and Social Deprivation indicators (MSD) for the social sub-dimension, the Material Deprivation indicators (MD), MSD or UK-Family Resource Survey indicators (FRS) for the cultural sub-dimension and the FRS for the mental sub-dimension. Table 5 provides an overview of the individual indicators or items that can be assigned to the individual social food poverty sub-dimensions.

**Table 5:** Indices and indicators used in poverty reports to assess social food poverty.

**Indicators**	**Social food poverty**		
	**Social**	**Cultural**	**Mental**
MD		Inability for the household to afford a meal with meat, chicken or fish or vegetarian equivalent every second day.	
MSD	Inability for the person to get together with friends/family for a drink/meal at least once a month.	Inability for the household to afford a meal with meat, chicken or fish or vegetarian equivalent every second day.	
FRS		Failing to afford to eat balanced meals.	Worry about whether food would run out before there was money to buy more.
		Skipping or cutting meals due to lack of money for food.	
		Eaten less than was considered right because there was not enough money for food.	

The *social sub-dimension* of social food poverty is covered in the poverty reports by the indicators of MSD, which is used in the EU-SILC and is a further elaboration of the (severe) MD indicators. Since 2014, 13 items have been surveyed annually in the EU countries and, in addition to the nine original material items, seven social items have been documented. One of the seven social items refers to the ability of individuals or households to get together with friends or family for a drink or meal at least once a month (51). This indicator therefore considers communal drinking or eating as a social activity, and thus the ability to do so regularly as an indicator of social participation. However, the indicator for social deprivation is based on an “enforced lack concept,” i.e., the person or household lacks the item for financial reasons [(51), p. 2]. It is therefore not possible to examine insights into the lack of social participation in food independently of material deprivation with this indicator. Material deprivation is always seen as a basic prerequisite and social deprivation in food as a possible outcome.

The *cultural sub-dimension* of food poverty is addressed by the MD/MSD and FRS indicators. The MD indicators measure the inability of individuals to afford certain goods that most people consider desirable or even necessary to live a decent life. The indicator measures the percentage of the population that cannot afford at least three out of nine goods, which include, among other holidays, a washing machine, a car and regular consumption of meat, fish, or a vegetarian equivalent (proteins). The indicator distinguishes between individuals who cannot afford the items on the list and those who do not have this item for another reason, e.g., because they do not want or need it (52). On one hand, this indicator reflects the scientific recommendations for balanced meals, but on the other, it also reflects what is culturally considered to be a balanced meal in Europe and which components are regarded as pertaining to it. According to the latest results, “(…) in 2020, 8.6% of the overall EU population and more than one in five people at risk of poverty were unable to afford a meal with meat, fish or a vegetarian equivalent every second day” (53). This signifies that nearly nine percent of the European population deviates from socio-culturally accepted diets and eating habits. However, it should be emphasized at this point that this is an element of the measurement of material deprivation. It likewise assumes that some individuals are excluded from key aspects of living conditions which appear to be customary across the whole EU due to a lack of monetary resources [(54), p. 11]. The indicator does not include other cultural elements and practices that determine participation in a country's food culture (such as the possibility to prepare festive meals on holidays or simply to have lunch with colleagues in the canteen) and it is further unable to identify potential resources that enable materially deprived individuals to maintain socio-cultural acceptable food patterns even when financial resources are lacking. The FRS of the UK Department for Work and Pensions collects items that also ask, based on material deprivation, about the ability to eat balanced meals and whether people in the household ever skip or cut back on meals because there are not enough financial resources for food. The indicators in the survey refer to food insecurity as a basic prerequisite for an active and healthy lifestyle. A healthy lifestyle thus includes socio-culturally accepted diets that are characterized by regularity, sufficiency, and balance. Individuals and households who do not regularly follow a diet described as balanced and who have few material resources are therefore considered to be food poor. The *mental sub-dimension* of social food poverty is rarely addressed. Only one of the items in the FRS food insecurity indicator asks whether individuals or households are worried about running out of food. There is no record of whether the diet also provides people experiencing poverty with pleasure, emotional security, balance, or self-esteem, or whether they can also be classified as deprived in this respect. Bizarre coping strategies, such as those recorded in food (in)security indices, are also not recorded in poverty reports. In summary, the significance of social food poverty in the poverty reports is thus limited to one indicator in each dimension. For the social dimension, the focus is on social participation through food, for the cultural dimension, on the possibility of following nutrition patterns that are culturally considered “healthy,” and for the mental dimension, on the concern for sufficient food.

## 5. Discussion

This study examined the various food (in)security and poverty indices and indicators at the global, European, US and UK level that show relations to various dimensions of a theoretical food poverty concept. The aim was to identify, first, which dimensions of food poverty are captured by these indices and indicators, and second, to examine how social food poverty is conceptualized and captured by them.

Overall, it can be concluded that aspects of food poverty are assessed by food (in)security as well as by general poverty indices and indicators. However, the indices and indicators in the food (in)security assessments are much more diverse and multidimensional than the indices and indicators used in general poverty assessments. The food (in)security indices are also directly related to nutrition and food, whereas the poverty reports only marginally contain items that refer to food or nutrition. A common feature shared by both types is that they each presuppose material deprivation as a prerequisite for the expression of the individual food poverty sub-dimensions (37, 55). While food (in)security is a relatively well-defined concept and the respective indices are usually based on the dimensions fanned out by official definitions, there are only few scientifically founded concepts for food poverty and accordingly no general definitions or concepts that are integrated as independent indices or indicators in the poverty reports. It is also important to note that, in contrast to food (in)security, there are no specific food poverty reports worldwide. There is also no official index of food poverty. The only attempt to develop a food poverty indicator was made in Ireland in 2012, based on data from the Annual Survey of Income and Living Conditions (SILC) (56). Ireland thus provides a first official step in measuring food poverty with a specific indicator, which could be extended to all countries surveying SILC data. The Irish food poverty indicator consists of three items (56, 57). In addition to the MSD, which has been assigned to the cultural dimensions of food poverty in our analysis (see Table 4), the Irish SILC also asks about the inability to afford a roast or vegetarian equivalent once a week. Moreover, the indicator includes whether there was at least 1 day in the last 2 weeks when the respondent did not eat a substantial meal due to lack of money. We assigned this item to the physiological dimension of food poverty, as it refers to periods of hunger caused by a lack of economic resources. These three items capture physiological and cultural dimensions of food poverty that are enforced by economic constraints. The example of the Irish food poverty indicator is given here because, on the one hand, it reflects the usual reporting on food poverty in poverty reports, but on the other hand, it also represents a hitherto unique attempt to capture the phenomenon and systematically highlight it as an area of poverty.

Furthermore, the Irish attempt to develop a food poverty indicator is also interesting and informative for the discussion of the meaning of social food poverty. Initially, the conceptualization of this indicator included an aspect of the social sub-dimension with the item of the inability for a person to get together with friends or family for a drink or meal at least once a month (56). However, this item was excluded after analyzing the available data, as the social class and income profile of those reporting the deprivation item did not seem to fit. In analyzing the data, Carney and Maître (56) focused on analyzing how each item was associated with other indicators of material deprivation, how dominant each item was, and also compared the reporting social classes and income profiles. The item was finally excluded from the food poverty indicator because, compared to the other items, when asked whether it is possible to invite family and friends over for a meal or a drink, the top two income quintiles reported a comparatively higher rate (six percent). The income and social class profile of those reporting the family and friends item differed from the profile of those who reported the other food deprivation items. This suggested that the population groups reporting the family and friends' item and the other food deprivation items were different and experienced a different form of deprivation. The correlation of the individual items also showed that only the “family and friends” item, i.e., the social sub-dimension of food deprivation, was determinant for this social class and occurred less in combination with the other items [(56), p. 22]. Therefore, the authors of the Irish Food Poverty Indicator did not consider this item as an appropriate means of measuring food poverty.

The underlying dilemma seems to be that social food poverty is seen exclusively as a consequence of economic deprivation. However, economic indicators can be a first starting point to approach potentially vulnerable groups, but they do not necessarily indicate whether and to what extent a household or person suffers from food insecurity (32). This raises the question of whether social food poverty can be considered and measured as a separate dimension or independent of income deprivation. Therefore, it might be enlightening to develop indices or indicators that focus on the social dimension. The following examples illustrate the necessity to consider social deprivation in food and nutrition as a serious phenomenon that can also be detrimental without economic deprivation: Individuals and households with high incomes can also be affected by social food poverty, e.g., career-oriented people who have not maintained private contacts due to lack of time, are socially isolated and have no one to go out with or who invites them to festive occasions. Another example concerns the physiological aspect, which can have an impact on social food poverty (58). More and more people suffer from food intolerances and allergies, which can also be affected by social exclusion, lack of cultural participation and mental health challenges (59). An invitation to a friend's home, a Christmas dinner with colleagues or even a holiday seems to become almost impossible or goes hand in hand with severe restrictions for those affected by circumstances that can trigger social poverty. In the reviewed food (in)security and poverty indices and indicators, social food poverty is always set in relation to material food poverty and especially economic deprivation. In each case, only one or two additional items are recorded for social food poverty dimensions and related to economic deprivation [see Poverty reports in our sample, like (60–63)]. However, this is not sufficient to fully capture social food poverty and to explain its dependencies. Food poverty cannot only be measured objectively and in a generally comparable way, but deprivation is also always experienced subjectively. Individuals who objectively still have sufficient financial resources for food expenditure but cannot feed themselves adequately for other reasons need to be better understood and appropriate representative data collected.

A measurement tool that comprehensively captures social food poverty and with which it can also be assessed independently of material deprivation appears very promising. Especially regarding food and nutrition, deprivation can be inadequately defined only with material poverty as the basis and solely being relevant when indicated by economic deprivation. Economic indicators can show effects on the material level of nutrition. For example, disposable income for food can limit access to food and lead to a reduction in the quality and variety of food, the reduction of quantities to skipping meals and experiences of hunger (17). However, the material measure of income poverty is not sufficient to speak in general terms of food-deprived individuals or households. Thus, the indicators used so far in food (in)security and poverty assessment are necessary but are incomplete and unable to capture the complexity of food poverty at different levels, in various dimensions and over time. Furthermore, the indicators within the different dimensions refer to various levels and stages of food poverty. Some refer to the conditional variables and risk factors (e.g., economic, and physical access to food, safe food, and clean drinking water), some to the consequences (e.g., physiological health, nutritional status, mortality, or socio-cultural exclusion) others try to capture the drivers and dynamics over time (e.g., food price volatility). Overall, it becomes evident that the assessment of food (in)security also sets different priorities within the individual dimensions of food poverty than the assessment of poverty in relation to food and nutrition. In addition to the economic dimension, other material as well as social dimensions and characteristics affect the manifestation of food poverty, which have so far received less attention in the indices and indicators of the poverty reports. The individual indices and indicators could therefore complement each other to a certain extent, not only to expand the concept of food poverty theoretically and conceptually, but also to provide richer and more holistic data and explanations for the phenomenon by changing the assessment bases and constellations. The food (in)security indices and indicators provide some evidence that, for example, physical food environments can also have a significant impact on the physiological dimension, as well as on the cultural and mental dimensions. Intra-household inequalities can also affect access to food within families, and thus the physiological dimension. Similarly, social coping strategies included in food (in)security indices can both serve as first indications of food poverty and provide knowledge for overcoming food poverty that may not (yet) be materially manifested.

Even if the food (in)security indices and indicators already include social and cultural aspects as well as mental aspects, and even if the poverty reports already cover a few manifestations of social food poverty, this is still insufficient to capture the complexity of the phenomenon. The focus is on measuring economic vulnerability and, regarding the social dimensions, on short-term coping measures to meet basic food needs. As a result, it is not possible to highlight the social, cultural, and mental drivers and consequences and the complex interactions of individual deprivation dimensions. Whether individuals or households can still eat out, invite friends, and maintain social relationships is not assessed. Likewise, resources in the social dimensions to overcome food poverty are not captured and thus do not highlight how different manifestations of the phenomenon of food poverty can be experienced despite material deprivation. Only the indicators used in the UK food security report and in the FSS include data on households that continue to gain access to food, for example through social policy or civil support (e.g., through food banks or community programs). This socio-political support has not yet been included as a separate sub-dimension in the theoretical food poverty concept, but it should be included under an additional political dimension together with other indicators which can be regarded as explanatory factors for the characteristics of food poverty.

A final important aspect to be discussed is that poverty in affluent societies has different manifestations, complex causes, and individual consequences than in countries of the Global South, where indices and indicators of food (in)security are often applied (64). Due to the strong socio-cultural character of the phenomenon of food and nutrition, a simple application of unidimensional measurements must be considered insufficient to make valid statements about the extent and the conditioning factors of food poverty at the level of various societies. Poverty always includes a subjective side, which can be experienced in combination or independently of material poverty and can have an impact on individual wellbeing and health in the social and cultural sphere (65, 66). Food and nutrition as practical life activities involve central social participation mechanisms (67) and poverty in food and nutrition can also be accompanied by deprivation in other areas of daily life (24).

## 6. Conclusion

The aim of this review and conceptual analysis was to advance the knowledge and practices of food poverty measurement by highlighting current priorities and emphasizing previously neglected dimensions. It has been discussed that the compilation of different indices and indicators from food (in)security and poverty assessments seems promising to obtain a more holistic understanding of food poverty and to expand the theoretical concept with further aspects within the individual sub-dimensions and with an additional political sub-dimension. Furthermore, it was argued that it appears necessary to relate the individual sub-dimensions independently of the economic sub-dimension to capture food poverty at an early stage and also without material deprivation as a necessary condition.

This article is the first attempt to take stock of what can currently be gathered in terms of indices and indicators in food (in)security and poverty assessment and to apply them to the theoretical concept of food poverty. For comprehensive coverage of all available and applied indices and indicators that can in some ways provide information on dimensions of food poverty and their possible manifestations, it is necessary to examine further areas. For example, regarding correlations between economic, physiological, social, and cultural dimensions, the area of nutrition monitoring is also relevant, and regarding correlations with the mental dimension, health monitoring. Here, too, it may be possible to find indices or indicators that can provide indications of aspects and manifestations of individual food poverty dimensions. Furthermore, all indices and indicators that have been developed in the scientific literature on the various dimensions of food poverty but have not yet been tested for their broad applicability must be systematically recorded and tested. Similarly, the qualitative research studies that highlighted different manifestations of social food poverty in a context-specific way need to be screened for potentially relevant indicators to expand the sub-dimensions of social food poverty.

Regarding the indices and indicators reviewed, it can be noted that the current practice of assessing and reporting on food poverty is characterized by defining food poverty through the application of certain indices or indicators of food (in)security and general poverty assessment, rather than defining the concept of food poverty before it is measured. To develop effective prevention and health promotion as well as policy approaches and to understand poverty in all its forms as well as its manifestations in the field of food and nutrition at different levels, it is essential to take a comprehensive concept of food poverty as a foundation. It is indispensable to understand the manifestations of food poverty more comprehensively, as well as its determinants and successful coping strategies, to systematically contribute to poverty alleviation and enable holistic healthy diets for all. The axiom “what gets measured gets managed” (68) or its opposite “what is not measured often gets ignored” (69) should not hinder efforts to achieve Sustainable Development Goal 1 (SDG1)—“no poverty” and SDG2—“zero hunger” at the international level and in countries of the Global North and should receive more scientific and operational attention (70).

## Data availability statement

The original contributions presented in the study are included in the article/supplementary material, further inquiries can be directed to the corresponding author.

## Author contributions

Conceptualization, supervision, and resources: TB. Analysis: SJ, EK, and TB. Investigation: SJ and EK. Writing—original draft: TB and SJ. Writing review and editing: TB and JY. All authors listed have made a substantial, direct, intellectual contribution to the work, and approved the manuscript for publication.
