# Corrigendum: A scoping review of the social dimensions in food insecurity and poverty assessments

**DOI:** 10.3389/fpubh.2024.1490591

**Published:** 2024-10-08

**Authors:** Tina Bartelmeß, Sarah Jasiok, Elias Kühnel, Juliane Yildiz

**Affiliations:** ^1^Faculty of Life Sciences: Food, Nutrition, and Health, University of Bayreuth, Kulmbach, Germany; ^2^Faculty of Law and Economics, University of Bayreuth, Bayreuth, Germany; ^3^Faculty of Agricultural, Nutritional and Environmental Sciences, Justus-Liebig-University Giessen, Giessen, Germany

**Keywords:** food poverty, food security, food insecurity, social dimensions, indices, indicators

In the published article, there was an error in Table 2 [Food (in)security and poverty reports indices and indicators referring to food poverty dimensions.] as published [the index “FIES” was missing in the row for “mental” and the column “Food (in)security indices^*^”]. The corrected Table 2 appears below.

**Table 2:** Food (in)security and poverty reports indices and indicators referring to food poverty dimensions.

**Dimension**	**Indicators**	**Description**	Food (in)security indices^*^	Poverty reports indices and indicators^**^
**Material food poverty**				
Economic	Food affordability	Food price levels, stability, and shocks, local cost of food required to meet a common energy intake	FAI; GFI; GFSI; UK-FSR	CPI
	Household-income	Lack of monetary resources to purchase food; share of income spent on food	CARI; FEI; FIES	MD; MSD
	Unconventional food income source strategies	Coping strategies to get money for food	CSI; FSS; LCS-FS	
Physical	National availability of food	Food supply, supply disruption, domestic production (export), food import dependence, food loss/waste; available food diversity/quality	Food-EPI; FSI; GFSI; HEI; MLDS	
	Food environment	Proximity to grocery stores; access to a reliable food/water source	FEI; GFSI; UK-FSR	MDI; MPI
	Food distribution	Quality of the road infrastructure	FSI	
Physiological	Malnourishment	Prevalence of undernourishment (stunting, wasting), overnutrition	FSI; GHI; GFI	BMI; MPI
	Diet quality	Healthy and nutritious food, dietary diversity, adequacy of micronutrient intake, consumption levels	DQI-I; FIES; Food-EPI; FSI; GFI; GFSI; HDDS; HEI; HFIAS	
	Food quantity	Consumption of different food groups, situations of hunger	CARI; FCS; HHS	
	Diet-related health outcomes	Diabetes, obesity, disability-adjusted-life-years, mortality rates	FSI; GFI; GHI	MPI; SPI
Hygienic	Food safety	Access to safe food and drinking water	GFI; GFSI	MPI; MPM; SPI
**Social food poverty**				
Social	Social integration	Getting together, eating out		MSD
	Communal networks	Coping behaviors (e.g., sending children to eat with neighbors); unconventional food sources (e.g., borrowing food from neighbors)	CARI; CSI; LCS-FS	
	Social food access barriers	Gender inequality in household food access, free institutional meals	CSI; FSS; GFSI; UK-FSR	
Cultural	Food customs and practices	Deviant food patterns, dietary change	CARI; CSI; FSS; HFIAS; HHS	FRS; MD; MSD
Mental	Worries about food	Uncertainty or concerns about insufficient food procurement	FIES, HFIAS; LCS-FS	FRS
	Bizarre coping strategies	Illegal income activities (theft, prostitution) due to lack of food, begging or scavenging for food	LCS-FS	

^*^CARI, consolidated approach for reporting indicators of food security; CSI, coping strategy index; DQI-I, diet quality index-international; FAI, food affordability index; FCS, food consumption score; FEI, food environment index; FIES, food insecurity experience scale; Food-EPI, food environment policy index; FSI, food sustainability index; FSS, food security supplement; GFI, global food index; GFSI, global food security index; GHI, global hunger index; HDDS, household dietary diversity scale; HEI, healthy eating index; HFIAS, household food insecurity access scale; HHS, household hunger scale; LCS-FS, livelihood coping strategies-food security; MLDS, market-level food diversity score; UK-FSR, UK-food security report indicators.

^**^BMI, body mass index; CPI, consumer price index; FRS, UK family resource survey indicators; MD, material deprivation indicators; MDI, multidimensional deprivation index; MPI, multidimensional poverty index; MPM, multidimensional poverty measure indicators; MSD, material and social deprivation indicators; SPI, social progress index.

In the published article, there was an error in Table 3 [Food (in)security indices with indicators on food poverty dimensions.] as published [a cross was missing in the row for “FIES” and the “Mental” column. Accordingly, the total value of the “mental” column has changed from 2 to 3]. The corrected Table 3 appears below.

**Table 3:** Food (in)security indices with indicators on food poverty dimensions.

**Food (in)security indices**			**Material food poverty**				**Social food poverty**		
**No**	**Index/ indicator**	**Name**	**Economic**	**Physical**	**Physiological**	**Hygienic**	**Social**	**Cultural**	**Mental**
1	CARI	Consolidated approach for reporting indicators of food security	x		x		x	x	
2	CSI	Coping strategy index	x		x		x	x	
3	DQI-I	Diet quality index-international			x				
4	FAI	Food affordability index	x						
5	FCS	Food consumption score			x				
6	FEI	Food environment index	x	x					
7	FIES	Food insecurity experience scale	x		x				x
8	Food-EPI	Food environment policy index		x	x				
9	FSI	Food sustainability index		x	x				
10	FSS	Food security supplement	x				x	x	
11	GFI	Global food index	x		x	x			
12	GFSI	Global food security index	x	x	x	x	x		
13	GHI	Global hunger index			x				
14	HDDS	Household dietary diversity scale			x				
15	HEI	Healthy eating index		x	x				
16	HFIAS	Household food insecurity access scale			x			x	x
17	HHS	Household hunger scale			x			x	
18	LCS-FS	Livelihood coping strategies-food security	x		x		x		x
19	MLDS	Market-level food diversity score		x					
20	UK-FSR	United Kingdom food security report 2021: theme 4: food security at household level	x	x			x		
**Σ**			10	7	15	2	6	5	3

In the published article, there was an error regarding a reference citation

A correction has been made to **3 Material and methods**, “*3.3 Inclusion and exclusion criteria*”. This sentence previously stated:

“To be included in the scoping review, the indices and indicators had to describe at least one aspect that can be assigned to a dimension of food poverty according to 25 concept.”

The corrected sentence appears below:

“To be included in the scoping review, the indices and indicators had to describe at least one aspect that can be assigned to a dimension of food poverty according to Feichtinger's (37) concept.”

In the published article, there was an error regarding the numbers stated

A correction has been made to **4 Results**, “*4.1 Dimensions of food poverty covered by identified indices and indicators*”, paragraph 2. This sentence previously stated:

“In comparison to social food poverty, which is considered by a total of eight indices, it becomes clear that the focus of the measurement of food (in)security is on the indicators that can predominantly be used to describe the status of material food poverty. Of the eight indices that also have indicators for social food poverty dimensions, six include indicators for the social dimension, five for the cultural dimension, and two for the mental dimension of social food poverty.”

The corrected sentence appears below:

“In comparison to social food poverty, which is considered by a total of nine indices, it becomes clear that the focus of the measurement of food (in)security is on the indicators that can predominantly be used to describe the status of material food poverty. Of the nine indices that also have indicators for social food poverty dimensions, six include indicators for the social dimension, five for the cultural dimension, and three for the mental dimension of social food poverty.”

In the published article, there was an error regarding the numbers stated.

A correction has been made to **4 Results**, “*4.2 Social dimensions of food poverty in food (in)security and poverty assessments*”, paragraph 1. This sentence previously stated:

“In total, five indices could be identified that show references to the social dimension of food poverty.”

The corrected sentence appears below:

“In total, six indices could be identified that show references to the social dimension of food poverty.”

In the published article, there was an error regarding the numbers stated and some information was missing.

A correction has been made to **4 Results**, “4.2 *Social dimensions of food poverty in food (in)security and poverty assessments*”, paragraph 1. This sentence previously stated:

“Furthermore, the *mental sub-dimension* of social food poverty is only addressed by two indices. The Livelihood Coping Strategies—Food Security Index (LCS-FS) records bizarre coping strategies to obtain food, such as begging for food or prostitution. The Household Food Insecurity Access Scale (HFIAS) includes the item of worrying about having enough food.”

The corrected sentence appears below:

“Furthermore, the *mental sub-dimension* of social food poverty is only addressed by three indices. The Livelihood Coping Strategies—Food Security Index (LCS-FS) records bizarre coping strategies to obtain food, such as begging for food or prostitution. The Household Food Insecurity Access Scale (HFIAS) and FIES include the item of worrying about having enough food.”

The authors apologize for this error and state that this does not change the scientific conclusions of the article in any way. The original article has been updated.

